# Type II Diabetes Patients under Sildenafil Citrate: Case Series Showing Benefits and a Side Effect

**DOI:** 10.1155/2020/4065452

**Published:** 2020-05-09

**Authors:** Livia M. Zimmermann, Mauricio S. Baptista, João Paulo Tardivo, Maria A. Pinhal

**Affiliations:** ^1^Faculdade de Medicina Do ABC, Santo André, Brazil; ^2^Universidade de São Paulo, Department of Biochemistry, Chemistry Institute, São Paulo, Brazil

## Abstract

**Background:**

Diabetes mellitus is a highly prevalent disease with rapid universal growth. In 2013, there were already 382 million people with diabetes, and it is expected that by 2035, this number will double. Chronic hyperglycemia causes a series of biochemical and structural changes, especially in the eyes, kidneys, heart, arteries, and peripheral nerves, which usually leads to the progression of microvascular disease. Several literature reports showed that the chronic use of phosphodiesterase-5 inhibitors enhances the insulin sensitivity, improves the markers of endothelial function, and helps in the treatment of severe extremity ischemia and pulmonary hypertension. We aim to test the effect of sildenafil citrate (SC) as a glucose and microcirculation regulator in diabetic patients, paying special attention to the consequences of its use in the regulation of blood glucose level. *Case Presentation*. Two male patients, aged 53 and 73 years, with type II diabetes, using oral hypoglycemic agents and presenting pathology associated with microcirculation alterations and ischemia, were medicated daily with SC. Both patients presented a reduction in the glycemic level, requiring lower doses or no other oral diabetes medications. Patient 1, who presented diabetic foot, was treated in the ambulatory, and patient 2, with chronic obstructive pulmonary disease and consequent mild pulmonary hypertension, was treated in the office. In addition to the clinical improvement of foot wounds and dyspnea due to the increase in microcirculatory perfusion, hypoglycemic episodes were observed in both patients under SC. The patient with pulmonary hypertension experienced one severe hypoglycemia episode and had to be taken to an emergency room.

**Conclusion:**

Type 2 diabetic patients may benefit from the use of a phosphodiesterase-5 inhibitor in order to improve the microcirculatory perfusion as well as glycemic control. However, adverse side effects may involve hypoglycemia. Since off-label use of SC in patients suffering from microcirculatory alterations has increased recently, our results showed that more studies are needed to verify the prevalence of hypoglycemia episodes as well as it's possible physiologic mechanism.

## 1. Introduction

Diabetes mellitus (DM) is an epidemic global disease affecting a substantial amount of people in every country irrespective of its development status. In 2013, there were 382 million people diagnosed with DM, and it is expected that, by 2035, this number will reach ∼600 million [1]. One of the main consequences of DM is the chronic hyperglycemia, favoring nonenzymatic reactions between sugars and several other biomolecules such as peptides and proteins, phospholipids, and nucleic acids (Maillard reaction), forming advanced glycation end-products and causing chronic inflammation, which harm microcirculation networks, especially in the eyes, kidneys, heart, arteries, and peripheral nerves [2].

Being affected by intracellular hyperglycemia, endothelial cells become dysfunctional and stop controlling the glucose transport. Due to the decreased nitric oxide (NO) production and the thickening of the vascular endothelium, vasomotor control is lost, causing permanent changes to the microcirculation [3]. Reduced efficiency of microcirculation brings several nefarious consequences. Systemic arterial hypertension (SAH), pulmonary arterial hypertension (PAH), and chronic obstructive pulmonary disease (COPD) represents the progressive increase in pulmonary vascular resistance, leading to ventricular dysfunction and finally death [2, 3]. Peripheral artery disease (PAD), diabetic foot, osteomyelitis, and amputations are also frequent outcomes in diabetic patients [4].

Nitric oxide (NO) is a multifunctional molecule exerting central roles in complex signaling networks and having several activation and effector signaling targets. Especially relevant to this report is its action as vasodilator, through the activation of guanylate cyclase and production of cyclic guanosine monophosphate (cGMP), acting in smooth muscle cells and causing a potent vessel dilatation. NO also plays an important role in the systemic regulation of blood glucose levels and insulin metabolism. All of the mentioned regulations can be controlled by phosphodiesterase inhibitors, which extend the action of cGMP [5, 6].

Sildenafil citrate (SC) is an inhibitor of phosphodiesterase type 5, therefore being an important vasodilator [5–7]. The main indication of SC is for the treatment of male sexual dysfunction. It is also important in cases of extremity ischemia in autoimmune diseases such as lupus rheumatoid arthritis, Raynaud disease, and pulmonary hypertension [2, 3, 5–7]. It is known that both insulin and SC increase the synthesis of nitric oxide in tissues such as skeletal muscle, endothelium, beta cells, liver, and brain, therefore participating in the maintenance of glucose homeostasis.

Indeed, SC has been shown to improve glucose metabolism in diabetic patients [7, 8]. Daily usage of SC for three months was shown to enhance the insulin sensitivity and to improve the markers of endothelial function [9–11]. A mechanism has been proposed correlating improvement in the microcirculation with nitric oxide synthase (type III) activation, which becomes calcium independent upon phosphorylation by cyclic nucleotide-dependent protein kinases [12]. However, the consequences of the chronic use of SC to the glycemic control have not been properly exploited. In this study, we report a series of two patients that were not responding properly to their respective treatments by only using antiglycemic drugs. These drugs were substituted by SC, and the consequences were followed in two patients. The unique aspect of this publication is to demonstrate the possibility of improving both glycemic and microcirculation controls in diabetic patients by means of chronic use of phosphodiesterase inhibitors. We add an important information to the medical knowledge related to the hypoglycemic episodes connected to the chronic SC usage and how to avoid them.

## 2. Patient Information

### 2.1. Case 1

A 62-year-old male, married, retired, with type 2 diabetes for more than 15 years, background to rheumatoid arthritis and myocardial revascularization, with no history of using alcohol or tobacco, without neuropathy, feeling pain in right foot which showed signs of dry necrosis in the plate and in the digital pulp of the third finger, and partial necrosis in the hallux reported. He Presented with distal pulses in the finger extremities, without fever, heartbeat of 80/min fc, and Pa 13 × 9 and was attended at the Diabetic Foot Treatment Center with diagnosis of type II diabetes and ischemic diabetic foot. He presented with progressive and painful necrotic plaques in the right hallux and 3rd right foot pod. The color of the feet was purple with fuming appearance. In terms of background, the patient reported treatment with a rheumatologist at the *Hospital das Clínicas* in São Paulo due to arthritis of the hands and coronary stent placement for 6 months at INCOR-SP. He was being medicated with prednisone, clopidogrel, and metformin.

**Figure 1:**
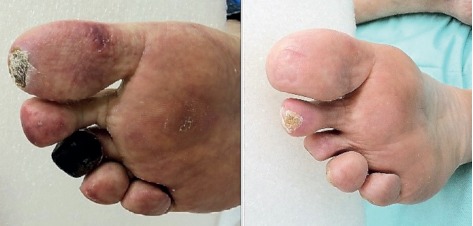
Images of the right foot before and after protocol with sildenafil citrate and PDT, showing the consequences of the treatment due to improvements in blood perfusion and wound healing. Methods and protocols have been described previously [4].

After clinical evaluation, he was medicated with Cilostazol 50, and an arterial echo Doppler of the lower limbs was requested. This examination concluded that the arterial trunks had vascular patency with three-phase flow. There was a minor, noticeable improvement of the right toes after two months of treatment with Cilostazol but with worsening of the contralateral toes. There were fixed cyanosis and resting pain of the 3rd and 4th toes of the left foot. Even an intensive care treatment by photodynamic therapy (PDT) did not improve the condition of the diabetic foot [4]. After consulting his cardiologist to authorize the use of SC, this drug was administered at the daily dose of 50 mg. This treatment was maintained for 10 months, with remission of the endpoint ischemia. The patient reported feeling well, and PDT started to respond well. After 5 PDT sections, feet lesion healed and the patient regained his colored feet and reported no pain (Figure 1).

One year after discharge, the patient returned again with lesion in his 1st left toe, ischemia in the 2nd right toe, with a necrotic plaque and ulcerated digital pulp, a lot of resting pain, and left lateral hallux ulcer. SC was once again administered, this time in doses of 50 mg every 12 hours (100 mg/day). After 3 months and 6 PDT sections, the 1st left toe had already healed, but the patient started to report hypoglycemia attacks. The use of Metformin and fasting was discontinued, as long as glucose levels were kept between 82 and 93 mg/dl, and SC was reduced again to 50 mg. The treatment of the 2nd finger, which was initially ulcerated, was finalized by PDT, and the patient was discharged.

During this relatively long period (more than two years), glycemia was obtained daily throughout several months, and average values are shown in Table 1. It is clear that SC allowed for a reduction and stabilization of glycemic values, with the extra benefit of having improved also the microvasculature, as indicated by the cure of the diabetic foot. It is also possible to note that there is an effect of the dose of SC, since during the 100 mg/day period, glycemia was significantly lower than in the other three periods in which the patient received a SC dose of 50 mg/day. However, as mentioned above, patient 1 under 100 mg/day of SC had an important hypoglycemic event, which was not observed at the 50 mg/day dose.

### 2.2. Case 2

A 73-year-old male, medical doctor, widower for 2 years, lives in the house with his two children. He is a social ethicist, smoker for 40 years, and quitted smoking 18 years ago as a response for the diagnosis of pulmonary emphysema, asthma, and systemic arterial hypertension (SAH). 10 years ago, he was diagnosed with Type 2 diabetes mellitus and dyslipidemia. For the long-term asthmatic status, he uses formoterol fumarate dihydrate 200 mg with bisoprolol hemifumarate 1.25 mg daily (inhaled bronchial dilator) 1x daily; for systemic arterial hypertension, losartan and hydrochlorothiazide 50/12.5 mg (morning and afternoon); for dyslipidemia, rosuvastatin 20 mg, AAS 100 mg.

Two months after starting SC, the patient reported having an important hypoglycemic attack (glucose level of 27 mg/dl) (Table 2). 3 hours after starting this episode, this patient still presented dysarthria, mental confusion, and loss of consciousness and was hospitalized in the ICU with good recovery after glucose replacement. The use of SC was suspended during the hospitalization period, and the medication for diabetes was resumed. After dismissal from the hospital, SC was reintroduced at a dose of 25 mg 3 times per day, and postprandial glucose remained around 138 mg/dl, even when consuming sweets; sulfonylurea (gliclazide) has been discontinued and home glycemia ranged from 101 to 117 mg/dl. Six months after the episode, the patient showed stabilization of symptoms with normal blood pressure, 61 beats per minute, 95% O_2_.

## 3. Discussion

One of the main consequences of chronic hyperglycemia is reducing the efficiency of microcirculation, which is possible to be enhanced by phosphodiesterase inhibitors, like SC. Interestingly, recent data indicate that SC also acts directly in the glycemic metabolism with similar effects of those of insulin. [8] If that is the case, the side effects of insulin administration, like hypoglycemia, could also exist expect in patients taking SC chronically. However, the reports available in the literature do not report on this important side effect.

Aiming to improve microcirculation status and to revert some of the consequences of DM, two of our patients were successfully treated with daily doses of SC. Our data indicate that SC may substitute glycemic control drugs for diabetic patients, since SC has unquestionable effects in terms of controlling glycemic levels besides improving the microvasculature. As a drug that mimics insulin, we should expect to have hypoglycemia events as one of the possible side effects of this treatment. Indeed, both of our patients reported hypoglycemia episodes. Therefore, our results agree with the benefits of using SC in diabetic patients but alert to the fact that more studies should be performed to better characterize the hypoglycemic effects of phosphodiesterase-5 inhibitors.

Several literature reports have shown the positive effects of SC in diabetes. In a randomized, controlled study using SC in prediabetic patients, Ramirez concluded that a 3-month regime of daily use of SC increased the insulin sensitivity, increased the glucose uptake through the muscle, and also increased the glucose-stimulated insulin release. A significant decrease in the inflammatory marker PAI-1 was also reported. [8] Other case reports have shown improvements in PAD-related diseases by the use of SC. [10, 11] No side effects related to the continued use of SC was reported, and safety issues restrained to care related to the known side effects of SC (patients using nitrites or with electrocardiographic abnormalities suspected of ischemic cardiovascular alterations).

In a recent study, it was observed that chronic and regular use of SC exerts beneficial action in diabetic animals, reversing the formation of superoxide by eroding the deleterious effects on the vascular bed caused by vascular dysfunction, thus increasing the reactivity of smooth muscle cells by the action of nitric oxide and increasing vascular homeostasis [9]. Interestingly, SC was shown to increase the pool of cGMP and eNOS activation and consequently increase in NO intracellular levels. This effect seems to occur through the activation of cGMP dependent protein kinase [12]. Therefore, the mechanisms by which SC mimics some of the effects of insulin in the glycolic metabolism are starting to be understood [8]. Accordingly, our results can be interpreted as another evidence that SC mimics the effects of insulin, including the decrease in the blood glucose level with possibilities to cause hypoglycemic episodes.

The DM patients whose clinical cases are here reported suffered either from diabetic foot or from chronic obstructive pulmonary disease. Both were given daily doses of SC, following clinical trials available in the literature, as well as indications of regulatory agencies in Brazil. Under SC, both patients improved from their medical conditions due to the control of problems related to the inefficient microcirculation and glycemic levels. However, both also showed important hypoglycemic attacks, which were alleviated by decreasing the SC dosage. An ongoing FDA multicenter, randomized, double-blind and placebo-controlled trial, aiming to definitively show the benefits of SC in PAD, has started and is expected to be concluded in 2020. The protocol is based in the use of a single morning 100 mg-SC dose [13], which is a prescription that can induce hypoglycemia attacks. Based in our experience, dividing the medication in several smaller doses during the day may help to avoid the hypoglycemia peaks.

## 4. Conclusion

SC undoubtedly improves peripheral circulation and consequently helps in the recovery of patients suffering from microcirculation complications. The improvement of two patients having, respectively, diabetic foot and pulmonary arterial hypertension agrees with the now-a-days trend of using SC in MD patients. The medical doctors responsible for treating these patients reported clear improvements in both patients, which were verified by lab results and the final disease recover. However, as expected from a drug that mimics some of the effects of insulin, hypoglycemic episodes are possible and even prevalent depending on the drug dose. Our experience indicates that dividing the SC daily dosage in smaller doses may help to avoid this important side effect [13].

## Figures and Tables

**Table 1:** Average glycemia of patient 1 under SC.

Period	Average glycemia (mg/dl)	SC
Oct/2013-Jun/2014	126 ± 39	None
Jun/2014-Jan/2015	96 ± 9	50 mg
Sep/2015-Dec/2015	84 ± 2	50 mg
Dec/2015-Mar/2016	79 ± 2	100 mg
Mar/2016-Aug/2016	87 ± 2	50 mg

**Table 2:** Behavior of fasting glycemia with and without the use of sildenafil and insulin.

Data	Use of SC	Home glycemia (mg/dl)
December/2015	(−)	90–210^*∗*^
November/2016	(−)	120–240^*∗*^
February/2018	(+)	90–250^*∗*^
February/2018, 15 days after start of SC	(+)	27^*∗∗*^

^*∗*^Maximum peaks for food abuse; ^*∗∗*^severe hypoglycemia.

## Abbreviations

cGMP: Cyclic guanosine monophosphate
COPD: Chronic obstructive pulmonary disease
DM: Diabetes mellitus
eNOS: Endothelial nitric oxide synthase
PAD: Peripheral artery disease
PAH: Pulmonary arterial hypertension
PDT: Photodynamic therapy
NO: Nitric oxide
SAH: Systemic hypertension
SC: Sildenafil citrate.
